# The podoplanin-CLEC-2 axis inhibits inflammation in sepsis

**DOI:** 10.1038/s41467-017-02402-6

**Published:** 2017-12-21

**Authors:** Julie Rayes, Siân Lax, Surasak Wichaiyo, Stephanie K. Watson, Ying Di, Stephanie Lombard, Beata Grygielska, Stuart W. Smith, Kassiani Skordilis, Steve P. Watson

**Affiliations:** 1 Institute of Cardiovascular Science, College of Medical and Dental Sciences, University of Birmingham, Edgbaston, B15 2TT UK; 2 Department of Renal Medicine, New Queen Elizabeth, Hospital Birmingham, Mindelsohn Way, Edgbaston, B15 2GW UK; 3 Department of Cellular Pathology, Queen Elizabeth Hospital Birmingham, Mindelsohn Way, Edgbaston, B15 2WB UK; 4 Centre of Membrane Proteins and Receptors (COMPARE), Universities of Birmingham and Nottingham, The Midlands, B15 2TT UK; 5 Institute of Microbiology and Infection, College of Medical and Dental Sciences, University of Birmingham, Edgbaston, B15 2TT UK

## Abstract

Platelets play a critical role in vascular inflammation through the podoplanin and collagen/fibrin receptors, C-type-lectin-like-2 (CLEC-2) and glycoprotein VI (GPVI), respectively. Both receptors regulate endothelial permeability and prevent peri-vascular bleeding in inflammation. Here we show that platelet-specific deletion of CLEC-2 but not GPVI leads to enhanced systemic inflammation and accelerated organ injury in two mouse models of sepsis–intra-peritoneal lipopolysaccharide and cecal ligation and puncture. CLEC-2 deficiency is associated with reduced numbers of podoplanin-expressing macrophages despite increased cytokine and chemokine levels in the infected peritoneum. Pharmacological inhibition of the interaction between CLEC-2 and podoplanin regulates immune cell infiltration and the inflammatory reaction during sepsis, suggesting that activation of podoplanin underlies the anti-inflammatory action of platelet CLEC-2. We suggest podoplanin-CLEC-2 as a novel anti-inflammatory axis regulating immune cell recruitment and activation in sepsis.

## Abstract (web summary)

Sepsis is a life-threatening condition where exaggerated inflammatory responses lead to severe tissue damage. Here, Rayes and colleagues show that the interaction between podoplanin and its receptor CLEC-2 on platelets plays a critical role in limiting inflammation during sepsis.

## Introduction

Sepsis is a life-threatening, severe systemic inflammatory response associated with multiple organ failure and death, which affects over 19 million patients annually^1,2^. Multi-organ failure results from excessive activation of complement, coagulation, and inflammation, making it likely that successful therapeutic intervention will require combination therapy^3,4^. In line with this, clinical trials targeting cytokines (e.g., anti-tumor necrosis factor (TNF)-α), bacterial virulence factors or coagulation factors have failed to show benefit in sepsis-mediated mortality^5^.

Therapeutic strategies targeting inflammation in sepsis must also preserve the role of the inflammatory reaction in mediating bacterial clearance. In peritonitis, clearance of bacteria depends on recruitment and activation of neutrophils and monocytes/macrophages^6–8^. Using a model of bacterial peritonitis combined with neutrophil depletion, it has previously been shown that while neutrophils regulate the early immune response, lacking them does not alter mortality, due to inflammatory macrophages increasing their anti-bacterial activity^7,9^. This has been shown to be due to the role of neutrophils suppressing monocyte activation via interleukin (IL)-10^10^. In contrast, combined monocyte and neutrophil ablation significantly increases bacterial load, inflammatory cytokines and septic lethality during cecal ligation and puncture (CLP)^10^. These data demonstrate a critical role for neutrophils and macrophages in regulating the severity of sepsis.

Platelets are now recognized to play a critical role in innate and adaptive immunity^11^. Platelets engulf and kill bacteria^12^, and protect against lipopolysaccharide (LPS)-induced septic shock by regulating macrophage function^13^. In a large cohort of septic patients, thrombocytopenia was associated with elevated levels of IL-8, IL-6, and IL-10, increased complement signaling, endothelial dysfunction, loss of vascular integrity, and increased mortality^14^. Thrombocytopenia is also associated with a poor outcome during infection in patients with sepsis-induced acute kidney injury (AKI)^15,16^.

Platelets regulate endothelial permeability and peri-vascular bleeding during inflammation and infection^17,18^. This protective role is predominantly mediated by the immunoreceptor tyrosine-based activation motif (ITAM)-containing receptors, CLEC-2) and glycoprotein (GP)VI^19,20^. Podoplanin, the only known endogenous receptor for CLEC-2, is widely expressed on a variety of epithelial surfaces, including kidney podocytes^21^ and alveolar type 1 cells^22^, fibroblastic reticular cells^23^, lymphatic endothelial cells^24^, and is upregulated on inflammatory macrophages^25,26^ and Th17 T cells^27^ during inflammation, and on tumor cells^28^. The GPVI ligands collagen and fibrin are present in the sub-endothelium and on luminal surfaces, respectively, and participate in platelet and leukocyte recruitment to the inflamed glomeruli^29^ and in thrombus formation and stability^30,31^.

Recently, we demonstrated a novel role for the podoplanin-CLEC-2 axis in driving thrombosis in liver during a mouse model of *Salmonella* infection^32^ and during sterile inflammation, in a mouse model of deep vein thrombosis (DVT)^33^. In these two models, podoplanin was shown to be up-regulated in the sub-endothelial wall and to become exposed at sites of breach thereby triggering platelet activation and thrombus formation, a process that has been termed thromboinflammation. Loss of platelet CLEC-2 prevents the inflammation-driven thrombosis in both models^32,33^. However, the role of CLEC-2 and podoplanin in the preceding inflammation is not known.

Based on the previously reported role of platelets during sepsis, and the upregulation of podoplanin on inflammatory macrophages, we hypothesize that CLEC-2 on platelets may also play a role in the early inflammatory events that gives rise to sepsis. In this study we have investigated this using two mouse models of sepsis, intraperitoneal (i.p.)-LPS and CLP. Using transgenic mouse strains, we demonstrate a protective role for platelet-expressed CLEC-2 in regulating the inflammatory reaction and preserving organ function in the two sepsis models. We further demonstrate that pharmacological blockade of the CLEC-2-podoplanin axis regulates the inflammatory reaction and immune cell infiltration during sepsis. Taken together these data suggest that the CLEC-2-podoplanin pathway plays a critical role in limiting inflammation during sepsis.

## Results

### Thrombocytopenia accelerates LPS-induced-organ damage

**Fig. 1:**
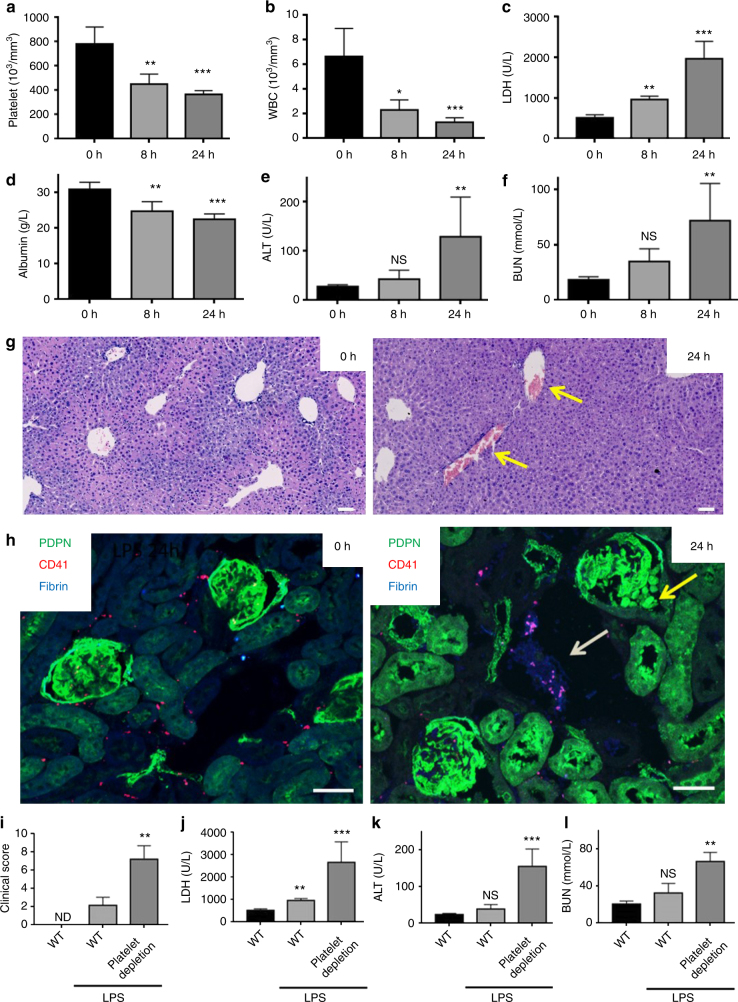
LPS-induced multiple organ damage is accelerated in platelet-depleted mice. Wild-type (WT) mice were injected with LPS (10 mg/kg) for 8 or 24 h. **a** Platelet and **b** white blood cell (WBC) count were measured. Blood serum biochemistry of **c** lactate dehydrogenase (LDH), **d** albumin, **e** liver enzyme alanine aminotransferase (ALT) and **f** blood urea nitrogen (BUN) (*n* = 5 in all groups) were measured. **g** Representative image of liver section stained with H&E (*n* = 5). Yellow arrows indicate thrombi. Scale bar = 100 μm. **h** Representative kidney section stained for podoplanin (PDPN), platelets (CD41) and fibrinogen/fibrin (*n* = 5). Yellow arrow indicates PDPN-positive cells in Bowman’s space and white arrow shows fibrin-platelet thrombi (purple). Bars = 50 μm. **i**–**l** Platelet depletion using rat IgG control or anti-mouse GPIbα antibody (1.5 μg/g) was performed 24 h before LPS injection. **i** Systemic clinical score, **j** LDH, **k** ALT and **l** BUN were measured in the blood 8 h post LPS (*n* ≥ 5 in all groups). Mean values shown with s.d. error bars. Differences between control groups and LPS-treated mice were assessed using Mann–Whitney *U*-test. **p < 0.01, ***p < 0.001

Previously published data demonstrate that LPS injection in mice increases the clinical severity of sepsis and induces thrombocytopenia, leukocyte infiltration, dysregulation of the coagulation cascade and thrombus formation in multiple tissues as well as sequential multiple organ failure^9,11,13^. We first confirmed that i.p. LPS induces thrombocytopenia and leukopenia (Fig. 1a, b) and progressively increases multiple organ damage (Fig. 1c). A decrease in liver and kidney function was observed 8 h post LPS that was further decreased after 24 h as measured by a hypoalbuminemia and an increase in plasma levels of sera alanine transferase (ALT) and blood urea nitrogen (BUN) (Fig. 1d–f). The decrease in liver and kidney function was associated with thrombus formation in multiple vessels (Fig. 1g, h). In the kidney, a network of fibrin and platelet-associated fibrin was observed in the glomeruli and peritubular capillary along with podocyte detachment in the Bowman’s space and adjacent tubules (Fig. 1h).

Platelets have been shown to protect against septic shock and to decrease organ damage in mice during LPS-induced sepsis^13^. In confirmation of this, reducing the platelet count below 30 × 10^9^/L by injection of an antibody to GPIbα resulted in an increase in clinical score (based on piloerection, reduced motor activity, increased lethargy and an increased incidence of an intermittent hunched posture; Supplementary Table 1) compared to isotype-treated controls (Fig. 1i). Moreover, thrombocytopenia accelerates organ function decline with a significant increase in LDH, ALT, and BUN 8 h post LPS compared to control mice (Fig. 1j–l). Thus our data confirm that platelets limit LPS-mediated sepsis and protect from multiple organ damage.

### Platelet CLEC-2 limits LPS-induced multiple organ damage

We next assessed the role of the platelet ITAM receptors CLEC-2 and GPVI in LPS-mediated organ injury using CLEC-2-deficient (CLEC2^fl/fl^PF4cre+), GPVI-deficient (GPVI^−/−^) and mice deficient in both receptors (CLEC2^fl/fl^PF4cre + GPVI^−^^/−^). In unchallenged mice, deletion of CLEC-2, GPVI or both receptors did not alter ALT or BUN level (Supplementary Fig. 1a, b). Double-deficient animals demonstrated a significant decrease in blood albumin levels compared to control mice indicative of mild changes in kidney filtration (Supplementary Fig. 1c). Moreover, deletion of CLEC-2, but not GPVI, impairs vascular integrity in the peritoneum as measured by hemoglobin levels in the peritoneal lavage of unchallenged mice (Supplementary Fig. 1d). This was also seen in double-deficient animals.

**Fig. 2:**
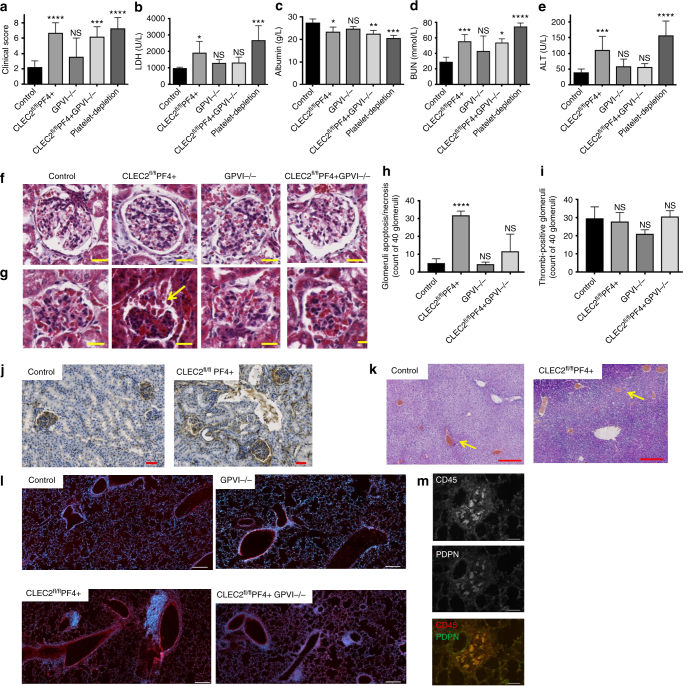
CLEC-2 deficient platelet exacerbates septic shock and multiple organ failure. Mice were injected with LPS (10 mg/kg) for 8 h and **a** clinical score was assessed. Blood serum biochemistry b LDH, c albumin, d BUN, e ALT (*n* ≥ 6 in all groups) were measured. Mean values shown with s.d. error bars. **f**, **g** Representative images of glomeruli in paraffin embedded kidney sections of unchallenged **f** or following 8 h LPS **g** wild-type (CLEC-2^fl/fl^), platelet CLEC-2 deficient (CLEC-2^fl/fl^PF4cre+), GPVI^−/−^ and double knock out (CLEC-2^fl/fl^PF4cre + GPVI^−/−^) mice using Masson’s trichrome stain. Scale bar = 20 μm. Arrow indicates the presence of epithelial cells and fibrin in Bowman’s space. **h** Histopathology score for epithelial cell sloughing in the Bowman’s space used as readout of necrosis/apoptosis and **i** the absence or presence of thrombi in the glomeruli expressed as means ± s.e.m. of 40 glomeruli from 5 mice in each group. Differences assessed using one-way ANOVA with Tukey’s multiple comparison tests ****p < 0.0001. **j** Representative images of paraffin embedded kidney cortex sections stained with anti-fibrinogen/fibrin antibody 8 h post LPS. Scale bar = 50 μm. **k** Representative images of paraffin embedded liver sections stained with H&E. Yellow arrows indicate thrombi bars = 200 μm. **l** Frozen lung sections stained for leukocytes (CD45) and nuclei (Hoechst 33342), Scale bar = 100 μm, or **m** CD45 and podoplanin. Scale bar = 50 μm. (*n* = 6 for all histology images)

Following LPS injection, a 30–50% drop in platelet count is observed in all mouse strains used, which was associated with leukopenia, suggesting that LPS-induced thrombocytopenia is independent of CLEC-2 and GPVI expression (Supplementary Fig. 2). Platelet-depleted, CLEC2^fl/fl^PF4cre+ and CLEC2^fl/fl^PF4cre + GPVI^−/−^, but not GPVI^−/−^ mice, showed a rapid and significant increase in clinical severity relative to controls in response to LPS, with a reduction in motor activity, increased lethargy and an increased incidence of an intermittent hunched posture observed (Fig. 2a).

The enhanced clinical severity observed in platelet-depleted and CLEC2^fl/fl^PF4cre+ mice was associated with a decrease in liver and kidney function at 8 h as assessed by LDH, blood albumin, BUN and ALT 8 h post LPS (Fig. 2b–e). The decrease in kidney function in CLEC2^fl/fl^PF4cre+ was associated with an increase in glomeruli congestion, apoptotic/necrotic podocytes and epithelial cells, red cells and fibrin deposition in the Bowman’s space (Fig. 2f–h). In contrast, no significant difference in fibrin deposition in the kidney was observed in CLEC2^fl/fl^PF4cre+ mice (Fig. 2i, j). Moreover, thrombus formation in the liver of CLEC2^fl/fl^PF4cre+ mice was not affected (Fig. 2k). This was consistent with the absence of podoplanin in the (peri-)vascular areas cells (Supplementary Fig. 3).

CLEC2^fl/fl^PF4cre + GPVI^−/−^ mice only demonstrated increases in blood albumin and BUN with LDH and ALT levels comparable to WT mice. Kidney histology indicated an intermediate phenotype with a reduction in glomerular and tubular injury compared to CLEC2^fl/fl^PF4cre+ mice (Fig. 2f–h). This suggests that GPVI deletion reverse the deleterious effect observed in CLEC2^fl/fl^PF4cre+ mice and that the degree of organ damage does not correlate with the degree of blood-lymphatic mixing or the level of hemoglobin in the peritoneal cavity, which are more severe in the double-deficient mice (Supplementary Fig. 1d).

In the lung, leukocyte infiltration was only observed around the bronchioles of CLEC2^fl/fl^PF4cre+ and CLEC2^fl/fl^PF4cre + GPVI^−/−^ mice following LPS injection, with a proportion of the infiltrating CD45+ cells expressing podoplanin (Fig. 2l, m).

Unchallenged CLEC2^fl/fl^PF4cre+ and CLEC2^fl/fl^PF4cre + GPVI^−/−^ have a mild reduction in platelet count compared to their littermate controls. To address whether the mild thrombocytopenia could account for the increase in severity in response to i.p.-LPS, we reduced the platelet count to ~350–600 × 10^3^/mm^3^ using an intermediate concentration of the GPIbα-depleting antibody. We observed no difference in clinical score, ALT or BUN levels compared to isotype-treated WT mice confirming that the mild thrombocytopenia does not underlie the increase in organ damage (Supplementary Fig. 4).

Taken together these results suggest that CLEC-2 deletion from platelets increases the severity of LPS-induced sepsis, which is accompanied by an increase in multiple organ injury.

### Podoplanin on podocytes does not alter LPS-induced AKI

**Fig. 3:**
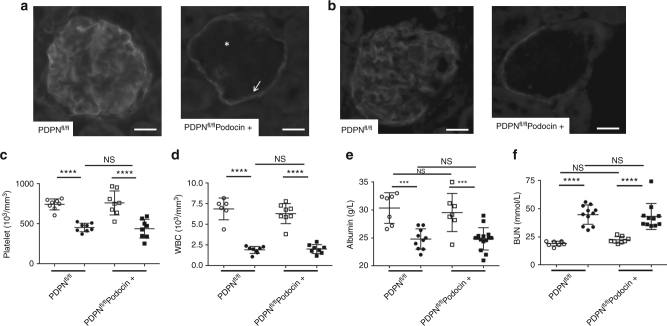
CLEC-2 protective effect is not mediated by podoplanin expressed on podocytes. Glomeruli on frozen kidney sections from PDPN^fl/fl^ mice and PDPN^fl/fl^Podocin-cre+ stained for podoplanin from **a** unchallenged and **b** LPS-treated mice (*n* = 6). Scale bar = 20 μm. Arrow indicates podoplanin staining on parietal epithelial cells and star indicates the absence of podoplanin on the podocytes. **c** Platelet count (*n* = 8), **d** white blood cell (WBC) count (*n* = 8), **e** blood albumin (≥7 mice) and **f** BUN levels in mice before (open symbol) (≥7 mice) and 8 h post LPS (closed symbol). Data are means ± s.d. Differences analyzed by one-way ANOVA with Tukey’s multiple comparison tests. ***p < 0.001, ****p < 0.0001

Podoplanin, the only known endogenous ligand for CLEC-2, is strongly expressed by renal podocytes. We used a podocyte-specific cre recombinase crossed to a floxed podoplanin strain, PDPN^fl/fl^Podocin-cre, to assess its role in the protective effect of CLEC-2 on glomerular function and structure. Genetic deletion of podoplanin on podocytes was confirmed by immunofluorescence (Fig. 3a, star), with podoplanin expression maintained on parietal epithelial cells (Fig. 3a, arrow). The level of podoplanin in control mice was slightly decreased in the glomeruli in response to LPS with depletion maintained in the PDPN^fl/fl^Podocin-cre+ mice (Fig. 3b). There was no change in platelet or white blood cell (WBC) counts, albumin or BUN in unchallenged or following LPS in the PDPN^fl/fl^Podocin+ mice (Fig. 3c–f). These data demonstrate that the protective effect of platelet CLEC-2 during LPS-induced sepsis is not associated with upregulation of podoplanin in glomeruli and is not mediated by podoplanin on renal podocytes.

### Platelet CLEC-2 limits inflammation during endotoxemia

Endotoxemia is known to increase pro-and anti-inflammatory cytokines in mice and human, in particular IL-10, IL-6, and TNF-α^34^. High expression of IL-10 and TNF-α correlate with poor prognostic in patients with severe sepsis whereas higher levels of TNF-α, IL-6, and soluble TNF receptor (sTNFR) are associated with early hemodynamic deterioration^35^. We therefore investigated the effect of platelet ITAM receptor deletion on cytokine and chemokine expression at 8 h following LPS challenge.

**Fig. 4:**
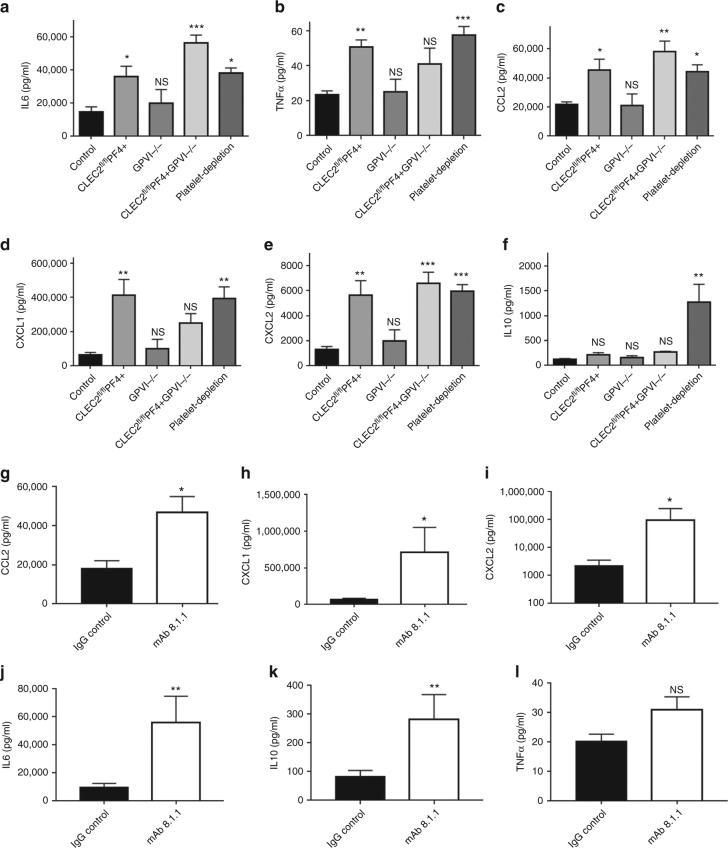
CLEC-2-podoplanin axis regulates the inflammatory reaction following LPS. **a**–**f** Mice were injected with LPS (10 mg/kg) for 8 h. Pro-inflammatory cyto-/chemokines **a** IL-6 **b** TNF-α, **c** CCL-2, **d** CXCL-1, **e** CXCL-2, and anti-inflammatory cytokine **f** IL-10 levels (pg/mL) measured in the blood 8 h post LPS from wild-type, platelet CLEC-2 deficient (CLEC-2^fl/fl^PF4cre+), GPVI^−/−^ and double knock out (CLEC-2^fl/fl^PF4cre+GPVI^−/−^) mice. Mean data shown ± s.e.m. (*n* = 7 for each group). Differences assessed using one-way ANOVA with Tukey’s multiple comparison tests. *p < 0.05, **p < 0.01, ***p < 0.001. **g**–**l** 2 h post LPS, α-PDPN (100 μg/mouse) or Syrian hamster IgG (IgG control) were injected i.v. into WT mice. Pro- and anti-inflammatory cyto-/chemokines **g** CCL-2, **h** CXCL-1, **i** CXCL-2, **j** IL-6, **k** IL-10 and **l** TNF-α levels (pg/mL) were measured 6 h later (i.e., 8 h post LPS). Differences between IgG control group and CLEC2^fl/fl^PF4cre+ was assessed using Mann–Whitney *U*-test (*n* = 7), NS non-significant, *p < 0.05, **p < 0.01

Deletion of CLEC-2 but not GPVI significantly increased serum TNF-α and IL-6, as well as the monocyte chemoattractant, chemokine (C-C motif) ligand 2 (CCL2), and the neutrophil chemokines, chemokine (CXC motif) ligand (CXCL)−1 and −2, compared to wild-type controls (Fig. 4a–e). The increase in IL-6, CCL-2, and CXCL-2 was also present in CLEC2^fl/fl^PF4cre + GPVI^−/−^ mice, whereas TNF-α and CXCL-1 were not significantly different from WT in the double-deficient strain, consistent with the intermediate phenotype described above.

The cytokine levels observed in LPS-treated CLEC-2^fl/fl^PF4cre+ mice were similar to those observed in thrombocytopenic mice post-LPS. In contrast, CLEC-2 deletion did not alter serum expression of IL-10 compared to LPS-treated wild-type controls (Fig. 4f). In order to assess whether the protective effect of CLEC-2 in the regulation of the inflammatory reaction is mediated by its interaction with its endogenous ligand, podoplanin, wild-type C57Bl/6 mice were injected with an anti-podoplanin blocking/crosslinking antibody, mAb 8.1.1, or isotype control 2 h post LPS. mAb 8.1.1 blocks podoplanin binding to CLEC-2 in vitro (Supplementary Fig. 5) and in vivo^33^ and induces crosslinking of podoplanin thereby potentially altering podoplanin signaling. Cytokine levels were measured in the blood 8 h post LPS. Disruption of the CLEC-2-podoplanin axis by administration of mAb 8.1.1 significantly increased expression CCL-2, CXCL-1, CXCL-2, IL-6, and IL-10 (Fig. 4g–k). No significant changes in TNF-α level was observed (Fig. 4l).

These data suggest that CLEC-2 deletion from platelets potentiates the acute inflammatory response induced by LPS. This dysregulated reaction is therefore a potential mechanism for the increased clinical severity observed in thrombocytopenic and CLEC2^flfl^PF4cre+ mice.

### CLEC-2 limits organ injury and inflammation during CLP

Injection of LPS reproducibly causes systemic activation of the innate immune system. However, sepsis in patients is far more complex triggered both by the infectious process and the immunological responses to microbial challenge^36–38^. Therefore, we used a bacterial model of peritonitis, CLP, to investigate the role of platelet CLEC-2 during sepsis.

**Fig. 5:**
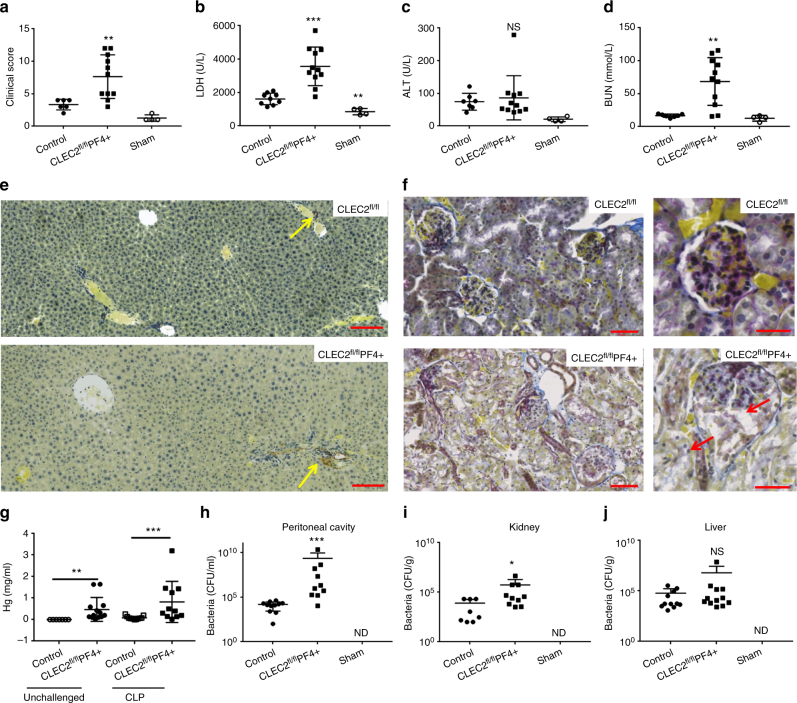
Platelet CLEC-2 limits bacterial dissemination and acute kidney injury during bacterial peritonitis. **a** CLEC2^fl/fl^PF4cre+ mice or littermate controls (*n* = 12) were subjected to cecal ligation and puncture (CLP) for 24 h and clinical score assessed. Cecum was exposed but no ligation or puncture performed in sham controls (*n* = 4). **b** lactate dehydrogenase (LDH), **c** liver enzyme alanine aminotransferase (ALT) and **d** blood urea nitrogen (BUN) were assessed 24 h post CLP. **f** Representative Martius Scarlet Blue-stained sections of renal cortex (*n* = 5). Collagen (blue), red blood cells/fresh fibrin (Yellow). Arrow shows cell detachment in the glomeruli and tubules.Scale bar = 50 μm. **e** Liver sections from control or CLEC2^fl/fl^PF4cre+ mice were stained for podoplanin (*n* = 5) (Brown, red arrow). Scale bar = 100 μm **g** Hemoglobin levels in the peritoneal cavity before and after CLP from WT and CLEC2^fl/fl^PF4cre+ mice. (Mean data shown ± s.e.m., *n* ≥ 6 for each group). **h** Bacteria in the peritoneal lavage fluid (PLF), **i** kidney and **j** liver homogenates were assessed post CLP. Colony unit formation (CFU) were counted and adjusted to the PLF volume or organ weight. Data are mean ± s.d. Differences between control group and CLEC2^fl/fl^PF4cre+ was assessed using Mann–Whitney *U*-test *p < 0.05, **p < 0.01, ***p < 0.001

As with the LPS-induced endotoxemia, a significant decrease in platelet and leukocyte count was observed in WT and CLEC2^fl/fl^PF4cre+ (Supplementary Fig. 6). Platelet-specific CLEC-2-deletion significantly increased the severity of CLP-induced sepsis (Fig. 5a) and tissue damage as measured by LDH level in the blood (Fig. 5b). No significant difference in liver ALT levels was observed (Fig. 5c). In line with this, no significant alteration in thrombus formation in the liver was observed, again consistent with the absence of podoplanin-positive cells around the vessels in the liver in this model (Fig. 5e). However, accelerated sepsis-mediated AKI was observed in CLEC2^fl/fl^PF4cre+ mice with increased BUN levels and severe glomerular damage with congestion and cellular necrosis/apoptosis associated with acute tubular injury (Fig. 5d, f).

Interestingly, bleeding in the peritoneal cavity of CLEC2^fl/fl^PF4cre+ mice did not significantly increase during CLP indicative of a similar loss in vascular integrity to WT controls (Fig. 5g). On the other hand, the increased severity of the clinical score and kidney damage was associated with an increase in bacterial load in the PLF and kidney 24 h after CLP compared to littermate controls (Fig. 5h, i). Bacterial load was not significantly different to controls in the liver, blood, spleen, or lung (Fig. 5j, Supplementary Fig. 7).

**Fig. 6:**
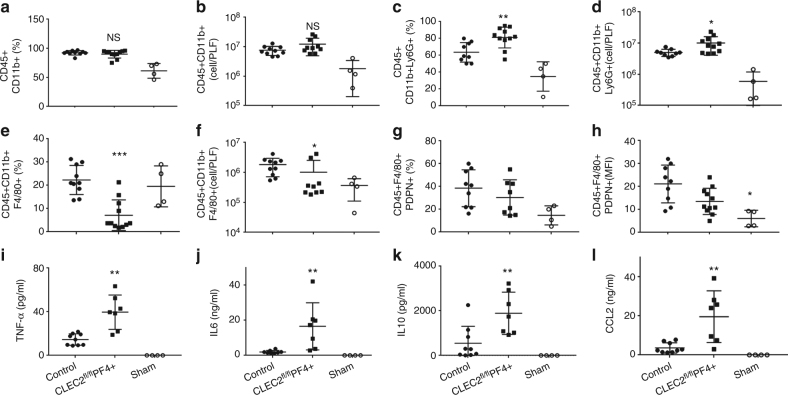
Platelet CLEC-2 regulates the inflammatory response during bacterial peritonitis. CLEC2^fl/fl^PF4cre+ or their littermate control mice following cecal ligation and puncture (CLP) for 24 h (*n* = 12). Immune cell recruitment to the peritoneal cavity was assessed by flow cytometry. **a** Percentage and **b** total number of myeloid cells (CD45+CD11b+) from CD45+ cells in peritoneal lavage fluid (PLF). **c** Percentage and **d** total number of neutrophils (CD45+CD11b+Ly6G+) in PLF. **e** Percentage and **f** total number of macrophages (CD45+CD11b+F4/80+) in PLF. **g** Percentage of macrophages expressing podoplanin, **h** Median fluorescence intensity (MFI) of podoplanin expressed on macrophages. **i** TNF-α, **j** IL-6, **k** IL-10, and **l** CCL-2 levels detected in the PLF. Data are means ± s.d. (*n* = 7 mice in CLP groups, *n* = 4 in sham group). Differences between control and CLEC2^fl/fl^PF4cre+ was assessed using Mann–Whitney *U*-test. *p < 0.05, **p < 0.01. ***p < 0.001

During this early stage of CLP, over 85% of the CD45+ recruited cells into the peritoneal cavity were myeloid cells, as shown by CD45+CD11b+ staining, with comparable numbers recruited in CLEC2^fl/fl^PF4cre+ mice and WT controls (Fig. 6a, b). However, a significant increase in the percentage and total number of neutrophils recruited was observed in CLEC2^fl/fl^PF4cre+ mice compared to WT controls (Fig. 6c, d). This was associated with a significant decrease in percentage and total number of macrophages recruited to the infected peritoneum in the CLEC2^fl/fl^PF4cre+ mice (Fig. 6e, f). Within this macrophage population, podoplanin was expressed at similar levels in WT and CLEC2^fl/fl^PF4cre+ mice, despite the reduction in actual numbers of macrophages recruited in CLEC2^fl/fl^PF4cre+ mice (Fig. 6g, h). An increase in TNF-α, IL-6, IL-10, and CCL-2 expression was also observed in the sera of CLEC2^fl/fl^PF4cre+ mice compared to their littermate controls following CLP (Fig. 6i–l).

Taken together these results suggest that platelet CLEC-2 regulates the severity of CLP by controlling the systemic and local immune response resulting in appropriate immune cell recruitment to the infected peritoneum, efficient bacterial clearance and limited organ damage.

### Podoplanin regulates macrophage recruitment during CLP

**Fig. 7:**
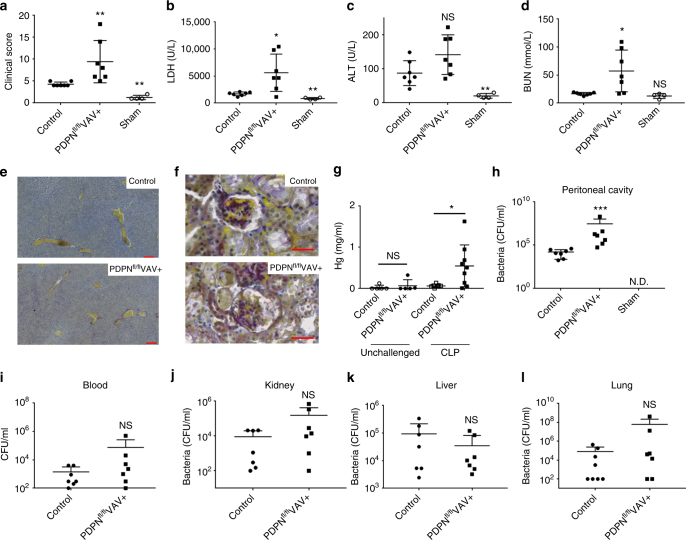
Podoplanin-deficient hematopoietic cells increase increase the severity of sepsis. PDPN^fl/fl^VAVcre+ or littermate control mice were subjected to cecal ligation and puncture (CLP) for 24 h and **a** clinical score assessed. **b** Lactate dehydrogenase (LDH), **c** alanine transferase (ALT), and **d** blood urea nitrogen (BUN) levels measured in the blood. Representative paraffin embedded **e** liver (Scale bar 200 μm *n* = 4) and **f** kidney (Scale bar 50 μm, *n* = 4) stained with Martius Scarlet Blue kit. Blue = collagen. Yellow = red blood cells/fresh fibrin. Red = old fibrin. Dark blue = nuclei. **g** Hemoglobin levels measured in the peritoneal lavage fluid (PLF). **h** Bacteria in the PLF, **i** blood, **j** kidney, **k** liver and **l** lung was assessed post CLP. Colony unit formation (CFU) were counted and adjusted to the PLF volume or organ weight. Data are means ± s.d. (7 mice in CLP groups, *n* = 4 in sham group). Differences assessed using Mann–Whitney *U*-test. *p < 0.05, **p < 0.01

Under inflammatory conditions, the activation of platelet CLEC-2 by podoplanin-expressing macrophages induces formation of platelet-macrophage aggregates and platelet-platelet aggregates in vitro and in vivo^25,26,32^. Using a mouse model of hematopoietic-deletion of podoplanin, PDPN^fl/fl^VAVcre+, a significant increase in sepsis severity and tissue injury was observed compared to littermate controls following CLP (Fig. 7a, b). CLP-induced thrombocytopenia and leukopenia in PDPN^fl/fl^VAVcre+ mice was comparable to WT (Supplementary Fig. 8), and no significant decline in liver function or thrombus formation was observed (Fig. 7c, e).

A significant increase in BUN levels in PDPN^fl/fl^VAVcre+ mice was associated with increased kidney injury compared to wild-type controls (Fig. 7d, f). In addition, a significant increase in bleeding within the peritoneal cavity was observed in PDPN^fl/fl^VAVcre+ mice compared to controls, indicating that podoplanin expressed on hematopoietic cells maintains vascular integrity following infection (Fig. 7g). Moreover, a significant increase in bacterial load in the peritoneal cavity of PDPN^fl/fl^VAVcre+ mice was observed compared to WT controls (Fig. 7h). However, no significant difference in bacterial dissemination to the blood, kidney, liver, or lung was observed in PDPN^fl/fl^VAVcre+ post CLP compared to WT controls (Fig. 7i–l).

**Fig. 8:**
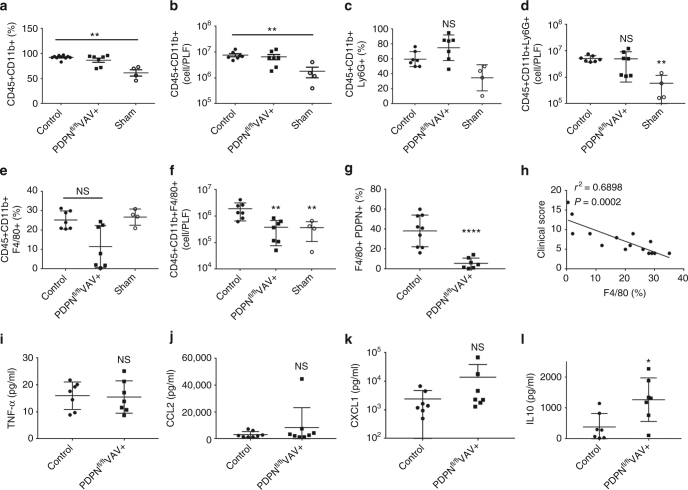
Podoplanin-deficient hematopoietic cells alter macrophage migration to the infected peritoneum. PDPN^fl/fl^VAVcre+ or littermate control mice were subjected to cecal ligation and puncture (CLP) for 24 h. Immune cell recruitment into the peritoneal cavity was assessed by flow cytometry. **a** Percentage and **b** total number of myeloid cells (CD45+CD11b+) in peritoneal lavage fluid (PLF). **c** Percentage and **d** total number of neutrophils (CD45+CD11b+Ly6G+) in PLF. **e** Percentage and **f** total number of macrophages (CD45+CD11b+F4/80+) in PLF. **g** Percentage of macrophages expressing podoplanin. **h** Correlation between percentage of macrophages recruited and clinical score. **i** TNF-α, **j** CCL-2, **k** CXCL-1, and **l** IL-10 levels detected in the PLF. Data are means ± s.d. (7 mice in CLP groups, *n* = 4 in sham group). Differences assessed using Mann–Whitney *U*-test. *p < 0.05, **p < 0.01, ****p < 0.0001

Recruitment of CD11b+-expressing myeloid cells to the inflamed peritoneum in PDPN^fl/fl^VAVcre+ mice was above sham controls but comparable to control post-CLP (Fig. 8a, b). No significant difference in neutrophil recruitment was observed post CLP in PDPN^fl/fl^VAVcre+ mice (Fig. 8c, d). However, the percentage and total number of macrophages recruited was reduced (Fig. 8e, f). Further, the percentage of macrophages expressing podoplanin is significantly reduced in PDPN^fl/fl^VAVcre+ mice, confirming deletion by the VAV1-driven cre recombinase (Fig. 8g). Although macrophage recruitment was altered in PDPN^fl/fl^VAVcre+ mice, the inflammatory status was largely unchanged during CLP as assessed by expression of TNF-α, CCL-2 and CXCL-1 (Fig. 8i–k). A significant increase was only observed in IL-10 expression in the PLF following CLP in PDPN^fl/fl^VAVcre+ mice compared to controls (Fig. 8l).

Podoplanin expression on macrophages in WT control mice varies post CLP (18–60%; Fig. 8g). In addition, the percentage of F4/80+ macrophages recruited was found to inversely correlate with the clinical score (Fig. 8h). We, therefore, tested whether podoplanin expression affects the phagocytic and bactericidal capacities of macrophages in vitro using bone marrow-derived macrophages (BMDM) isolated from PDPN^fl/fl^VAVcre+ and control mice. BMDMs were incubating in the presence or absence of LPS with *E. coli* bio-particles or with live *E. coli* CFT73 bacteria. No significant differences in *E. coli* bio-particle uptake or *E. coli* killing were observed (Supplementary Fig. 9).

Together these data suggest that podoplanin expression on hematopoietic cells plays a role in regulating macrophage recruitment to the infected peritoneum during CLP, modulating bacterial load in the peritoneal cavity and limiting subsequent organ damage.

### Podoplanin regulates inflammation in vitro and in vivo

Cytokines constitute a double-edged sword in infection; while immune cell recruitment and cytokine production is crucial to clear bacteria, excessive production can induce multiple organ damage^39–41^. In both LPS-induced and CLP-induced sepsis models, TNF-α level, as well as other cytokines, was significantly increased in platelet CLEC-2-deficient animals compared to their littermate controls. Interestingly, TNF-α levels were not altered in the presence of an anti-podoplanin antibody. TNF-α is expressed predominantly by neutrophils and inflammatory macrophages, and both platelets and platelet releasate have been shown to reduce secretion of TNF-α by macrophages in response to high dose LPS^13^. It has been previously shown using the RAW 264.7 macrophage cells that LPS upregulates podoplanin expression^26^. We therefore investigated whether podoplanin on macrophages modulates TNF-α secretion.

**Fig. 9:**
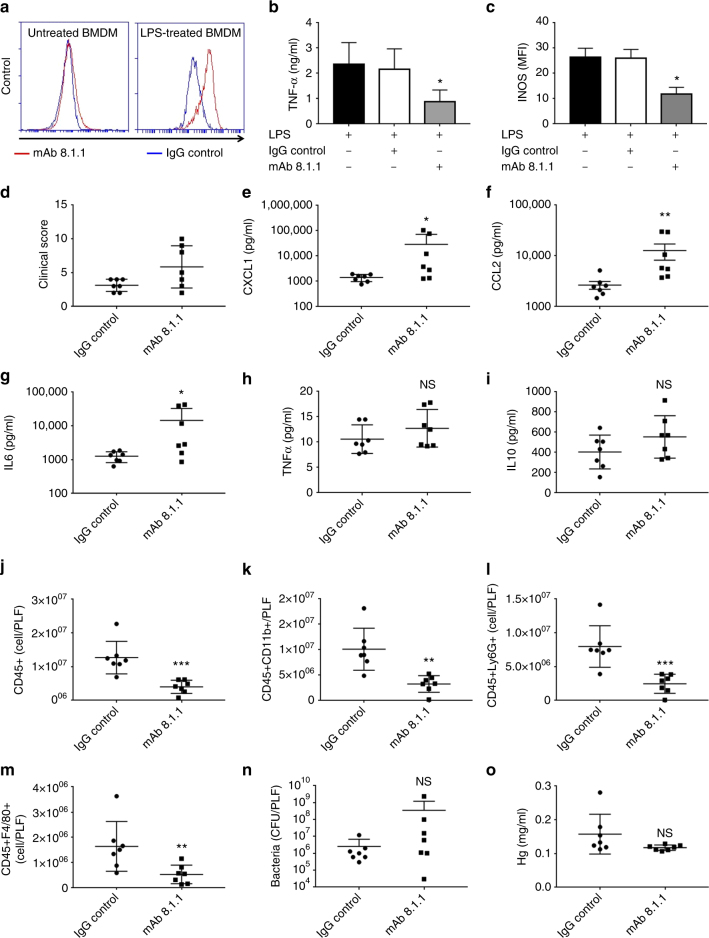
CLEC-2-podoplanin axis regulates the inflammatory reaction in vitro and in vivo. **a**–**c** Bone marrow derived macrophages (BMDM) were incubated in the presence of LPS (1 μg/ml) or media for 18 h. **a** Podoplanin expression assessed by flow cytometry gated on annexin V-negative BMDM. **b**,**c** LPS-treated BMDM were incubated with anti-podoplanin antibody (mAb 8.1.1) or Syrian hamster IgG (IgG control) for 24 h. **b** TNF-α secretion and **c** iNOS expression was assessed. **d**–**o** WT mice were subjected to cecal ligation and puncture (CLP) for 24 h. Two hours post cecal ligation and puncture (CLP), mAb 8.1.1 (100 μg/mouse) or Syrian hamster IgG (IgG control) were injected i.v. and **d** clinical score assessed at 24h. **e** CXCL-1, **f** CCL2, **g** IL-6, **h** TNF-α and **i** IL-10 were measured in peritoneal lavage 24 h after CLP. Immune cell recruitment to the peritoneal cavity was assessed by flow cytometry. Total number of **j** CD45+ cells **k** CD45 + CD11b+ myeloid cells, **l** neutrophils (CD45+CD11b+Ly6G+) and **m** macrophages (CD45 + CD11b + F4/80+) in PLF was measured. **n** Bacteria in the PLF was assessed 24h post CLP. Colony unit formation (CFU) were counted and adjusted to the PLF volume. **o** Hemoglobin levels measured in the peritoneal lavage fluid 24 h post CLP. Data are means ± s.d. (*n* = 7). Differences analyzed by Mann–Whitney *U*-test. NS non-significant, *p < 0.05, **p < 0.01, ***p < 0.001

In line with previous findings, we observed an upregulation of podoplanin on BMDM in the presence of high dose of LPS (1 μg/ml) (Fig. 9a). We used the anti-podoplanin mAb 8.1.1 to investigate the role of podoplanin in the regulation of TNF-α secretion from BMDM induced by LPS. mAb 8.1.1 blocks podoplanin binding to CLEC-2 in vitro (Supplementary Fig. 5) and in vivo^33^ and induces crosslinking of podoplanin thereby potentially altering podoplanin signaling. A significant decrease in TNF-α secretion from macrophages was observed in the presence of mAb 8.1.1 compared to controls (Fig. 9b). This was associated with a significant decrease in the pro-inflammatory M1 marker inducible nitric oxide synthase (iNOS) as assessed by flow cytometry (Fig. 9c).

As disruption of CLEC-2-podoplanin axis during endotoxemia partially mimics the phenotype observed in CLEC2^fl/fl^PF4cre+ mice, we assessed the role of anti-podoplanin antibody on the inflammatory reaction and immune cell recruitment during bacterial peritonitis. Wild-type, C57Bl/6, mice were injected i.v. with mAb 8.1.1(100 μg per mouse) 2 h post CLP. Injection of the anti-podoplanin antibody moderately increased the clinical severity (Fig. 9d). This effect was associated with an increase in CXCL-1, CCL-2 and IL-6 levels in the peritoneal cavity (Fig. 9e–g). The levels of TNF-α and IL-10 were not altered in the presence of the podoplanin antibody. Despite an increase in cytokines and chemokines, there was a significant decrease in the recruitment of CD45+CD11b+-expressing myeloid cells recruited to the peritoneum (Fig. 9j–m). Despite these changes, injection of the anti-podoplanin antibody did not significantly alter bacterial load or the amount of bleeding in the peritoneal cavity (Fig. 9n, o).

These data demonstrate that expression of podoplanin on inflammatory macrophages regulates the expression of TNF-α in vitro, with disruption of the CLEC-2-podoplanin pathway in vivo altering cytokine and chemokine expression and cellular recruitment during CLP. Together, these data confirm that the CLEC-2-podoplanin axis regulates the inflammatory reaction in vitro and in vivo.

## Discussion

In this study we demonstrate that (i) in two independent mouse models of sepsis, platelets limit the clinical severity via the platelet ITAM receptor, CLEC-2; (ii) this effect of CLEC-2 is independent of thrombosis; (iii) the protective role of CLEC-2 is partially mediated by its interaction with podoplanin expressed on inflammatory macrophages but not kidney podocytes; and (iv) the CLEC-2-podoplanin interaction regulates immune cells infiltration and the inflammatory reaction at the site of infection.

Platelets have been shown to play multiple roles in sepsis, with the platelet receptors CLEC-2 and GPVI having a critical role in the maintenance of vascular integrity at sites of inflammation^17–20^. In addition, platelet CLEC-2 triggers thrombus formation in the liver in mouse models of *Salmonella*-mediated infection^32^ and deep vein thrombosis^33^ through a process known as thromboinflammation. In both models, podoplanin is up-regulated in response to the inflammatory reaction and becomes exposed at sites of breach in the vessel wall leading to platelet activation and thrombus generation. In the present study, we show for the first time a role for platelet CLEC-2 in the regulation of the inflammatory reaction independent of an effect on thrombosis, showing that the podoplanin-CLEC-2 axis plays a role both in inflammation and in inflammation-driven thrombosis (thromboinflammation).

In this study, we demonstrate that the podoplanin-CLEC-2 axis plays a protective role in sepsis by controlling the cytokine/chemokine storm following infection limiting organ damage. In addition, platelet CLEC-2 regulates immune cell infiltration at the site of infection and promotes macrophage recruitment leading to the control of bacterial load in the infected peritoneum. In contrast, GPVI appears to have an opposing role: GPVI-deficient mice had a similar response to wild-type controls, with the double-deficient mice having an intermediate phenotype in regard to multiple organ damage relative to platelet-specific CLEC-2-deficient mice. Consistent with these results, a recent study by Pierre S et al.^42^, demonstrated that GPVI promotes the pro-inflammatory phenotype of macrophages during cutaneous inflammation. Whether GPVI, via its interaction with tissue collagen and/or fibrin, modulates the inflammatory reaction during sepsis by altering immune cell activation is the focus of ongoing studies.

Our data show a beneficial role of platelet CLEC-2 during sepsis that is independent of thrombosis, as there was no significant difference in thrombus formation in kidney or liver vessels in the absence of platelet CLEC-2. The absence of a role of CLEC-2 in thrombus formation is consistent with the lack of peri-vascular podoplanin expression which is currently the only known ligand for CLEC-2. In LPS- or CLP-induced sepsis models, inhibition of thrombus formation does not improve organ damage or survival^43,44^, further emphasizing that the present results are independent of a role of CLEC-2 in thrombus formation. Recently, neutrophil extracellular traps (NETS) were shown to promote intravascular coagulation and microvascular damage in sepsis^9,45^. These results, combined with ours, highlight the complex interplay of the inflammatory reaction and thrombosis in sepsis.

In both sepsis models, clinical severity was associated with a significant increase in organ damage, in particular AKI in the absence of platelet CLEC-2. This may be related to the early time points used given that the kidney is the first organ to show damage in septic patients, with death usually caused by AKI^46^. The CLEC-2 ligand, podoplanin is highly expressed by glomerular podocytes^21^. Nevertheless, using targeted deletion of podoplanin, we show that the protective role of platelet CLEC-2 is not mediated by podoplanin on podocytes. Rather, our data support a role for podoplanin on inflammatory macrophages in reducing the bacterial load in the peritoneum.

Bacterial dissemination, the cyto-/chemokine profile, and recruitment of neutrophils to the peritoneum differed in PDPN^fl/fl^VAV1cre+ compared to CLEC2^fl/fl^PF4cre+ mice during CLP. These data suggest that the underlying mechanism is not solely due to the interaction of podoplanin on hematopoietic cells and CLEC-2 on platelets and could therefore involve the interaction of podoplanin with additional ligands or podoplanin expressed on other cell types.

It is unclear whether the podoplanin-CLEC-2 axis limits sepsis through parallel mechanisms, namely (i) the activation of platelets; (ii) the interaction with podoplanin on inflammatory macrophages increasing platelet-macrophage aggregation and bacterial trapping, and (iii) controlling the inflammatory response via a macrophage-dependent mechanism and thereby allowing efficient macrophage recruitment to the site of infection. There are also a variety of cells that express podoplanin that could be involved including epithelial, stromal and lymphatic endothelial cells. The presence of multiple mechanisms for CLEC-2 and podoplanin in regulating macrophage functions and the presence of multiple cells is the focus of ongoing research.

In this study, we show that anti-podoplanin antibody, mAb 8.1.1, modulates the inflammatory reaction following sepsis. Our in vitro data demonstrate that podoplanin expressed on inflammatory macrophages regulates not only platelet aggregation, but also macrophage TNF-α secretion. In vitro, mAb 8.1.1decreases TNF-α secretion from LPS-treated BMDM. However, in vivo, injection of the anti-podoplanin antibody inhibits immune cell recruitment to the infected peritoneum despite an increase in pro-inflammatory cytokines and chemokines. Of note, relatively high concentrations of mAb 8.1.1 were required to block the interaction of podoplanin and CLEC-2 in vitro. This may explain the intermediate phenotype observed in vivo relative to that observed in platelet CLEC-2 deficient mouse models.

Importantly, the anti-podoplanin antibody regulates the inflammatory reaction and immune cell infiltration during sepsis without inducing bleeding in the peritoneal cavity. Thus, the anti-podoplanin antibody represents a novel way to modulate the inflammatory reaction and immune cell infiltration in a manner that is dependent on podoplanin expression. As inflammation is also required for efficient bacterial clearance, the selective modulation of the inflammatory system is essential to prevent an uncontrolled bacteraemia.

One potential limitation of this study is the use of PF4 cre to generate platelet-specific CLEC-2-deficient mice. A recent study has shown that the PF4-driven cre recombinase is expressed on a subset of immune cells and on a small subset of activated DCs in mesenteric lymph nodes following exposure to LPS^47^.

In conclusion, this study suggests a novel mechanism for the protective role of platelets in sepsis through the ITAM receptor CLEC-2. We demonstrate, in two mouse models of sepsis, that platelet CLEC-2 limits clinical severity of sepsis by potentially controlling multiple parameters including monocyte/macrophage migration to the site of infection, inflammatory mediator expression, limiting bacteremia and organ damage. These conclusions are based on the use of platelet-specific CLEC-2-deficient mice or by pharmacological inhibition of CLEC-2-podoplanin interaction using an anti-podoplanin antibody. Our study highlights a new role for platelets in regulating innate immunity during infection through the podoplanin-CLEC-2 axis.

## Methods

### Mice

All experiments were performed in accordance with UK laws (Animal [Scientific Procedures] Act 1986) with approval of local ethics committee and UK Home Office approval under PPL P0E98D513 and 70/8576 granted to the University of Birmingham. The following mice were used: the ITAM-deficient strains GPVI^−/−^, platelet CLEC-2^−/−^ (CLEC2^fl/fl^PF4cre+) and double-deficient (CLEC2^fl/fl^PF4cre + GPVI^−/−^), and mice with targeted deletion of podoplanin in cells of hematopoietic lineage, PDPN^fl/fl^VAV-iCre^48–51^. Mice expressing the Podocin cre^52^ were crossed with PDPN^fl/fl^ to generate the PDPN^fl/fl^Podocin+ strain. All mice were on a C57Bl/6 background. Wild-type (WT) C57Bl/6 mice were purchased from Harlan Laboratories (Oxford UK). For intraperitoneal injection of LPS, 12–16 weeks females and males (2:1 ratio) were used. For CLP, 12–16 weeks old females were used.

### LPS-induced endotoxemia

LPS (Escherichia coli 055:B5, Sigma) was diluted in pyrogen-free saline and injected intraperitoneally to sex and age matched mice. A clinical score severity was performed after 8 or 24 h based on each animal’s appearance, activity, response to stimulation and openness of their eyes (Supplementary Table 1). At 8 or 24 h post injection, blood was collected in ethylenediaminetetraacetic acid (EDTA) for blood cell counts or without anticoagulant using retro-orbital bleeding. Kidneys, livers and lungs were collected.

### Cecal ligation and puncture model

Cecal ligation and puncture (CLP) was performed as described previously^53^. Briefly, female mice were anaesthetized using isoflurane, the abdominal skin shaved, and under aseptic conditions, a 1 cm midline incision performed. The cecum was ligated with a silk suture and punctured once through-and-through with a 19 gauge needle. The cecum was then gently squeezed to exteriorize a drop of feces and returned to the peritoneal cavity. The laparotomy site was closed with 4.0 silk and animals returned to their cages. After 24 h, clinical score was assessed as previously described^54^ and blood, peritoneal lavage fluid (PLF), liver and kidneys collected. Peritoneal lavage fluid was collected using 2 ml ice-cold phosphate-buffered saline (PBS) supplemented with EDTA (10 mM). Hemoglobin was measured in PLF after lysing red cells as previously described^19^. Liver (right lobe) and one kidney were homogenized in ice-cold sterile PBS and the homogenates plated on lysogeny broth (LB) agar to determine the number of colony forming units (CFU). The other kidney was processed in paraffin for immunohistochemistry. Neutrophil count in the PLF was measured by flow cytometry using anti CD45, CD11b and Ly6G (clone1A8, eBiosciences) and macrophages analyzed using anti-F4/80 antibody (eBiosciences). Podoplanin expression was analyzed using antibody clone 8.1.1 (eBiosciences).

### Platelet depletion procedure

Platelets were depleted by i.p. injection of rat anti-mouse GPIbα antibody (1.5 μg/g for severe thrombocytopenia and 0.3 μg/g for mild thrombocytopenia; Emfret, Wurzburg, Germany) delivered 24 h before LPS injection. Rat IgG was used as an isotype control. Blood was collected in EDTA to measure the level of depletion using an ABX Pentra 60 blood counter (Horbia Ltd, Northampton, UK).

### Blood chemistry and serum cytokines and chemokines

For blood chemistry and cytokine measurements, whole blood was collected without anticoagulant, incubated for 1 h at 37 °C and serum isolated by centrifugation at 2000×*g* for 30 min. Blood urea nitrogen (BUN), blood albumin, lactate dehydrogenase (LDH), and alanine transferase (ALT) were measured on an AU680 clinical chemistry analyzer (Beckman Coulter, High Wycombe, UK) using reagents and settings recommended by the manufacturer. All cytokines and chemokines in the blood and PLF were analyzed using Fluorokine MAP Multiplex (R&D systems, Abingdon, UK).

### Histology and immunohistochemistry

For frozen sections, the renal capsule was removed and kidneys bisected. They were then fixed initially in 4% paraformaldehyde (PFA) for 2 h, subsequently transferred into 20% sucrose overnight and frozen in optimum cutting temperature (OCT) compound (Tissue-Tek, The Netherlands). For immunohistochemistry, kidneys were fixed overnight in 4% PFA, dehydrated and embedded in paraffin. Livers were snap-frozen in liquid nitrogen. Lungs were infused in situ, post-mortem with 1.5 ml of a solution containing OCT in PBS (1:2) introduced via the trachea using a 19G needle. The trachea was then sutured and lungs removed en bloc and frozen over dry ice. Lung and liver sections (12 μm) and kidney sections (6 μm) were processed for immunofluorescence staining or immunohistochemistry. Masson’s trichrome stain (Abcam, Cambridge, UK) and Martius Scarlet Blue stain (Atom Scientific, Hyde Cheshire, UK) were applied on paraffin sections. Anti-mouse podoplanin monoclonal antibody (mAb, clone 8.1.1), anti-mouse mAb CD45 (Clone 30-F11) (all from eBiosciences), anti-mouse mAb CD41 (Clone MWReg30, BD biosciences), and anti-fibrin/fibrinogen (no. YNGMFBG7,Accurate chemical and scientific Co, NY, USA) (all used at final concentration of 5 µg/ml) were used for immunofluorescence on frozen sections. Images were analyzed using a Zeiss confocal microscope or Zeiss Axio Scan.Z1 microscope and ZEN software or by confocal microscopy. Glomerular changes were assessed on blinded sections based on 40 glomeruli per kidney. Two histological features were evaluated as present or absent: glomerular congestion with or without thrombi and sloughing of epithelial cells in the Bowman’s space consistent with necrosis/apoptosis.

### Platelet and macrophage preparation and co-culture

Washed platelets were prepared as previously described^55^. Bone marrow-derived macrophages (BMDMs) were prepared as previously described with some modifications^56^. Briefly, bone marrow cells were plated at 10^6^ per ml in 20% L-929 cell conditioned media. After 7 days in culture, BMDMs were washed and activated with LPS (1 μg/ml) for 18 h. Cells were scraped and podoplanin expression assessed by flow cytometry on an Accuri C6 Plus (BD Biosciences, Oxford, UK) using PE conjugated anti-podoplanin antibody (mAb 8.1.1). For co-culture experiments, anti-podoplanin antibody (mAb 8.1.1) (10 μg/ml) or isotype control were added for 24 h in the presence of LPS (1 μg/ml). TNF-α in the supernatant was measured by ELISA (Peprotech, London, UK). The RAW 264.7 macrophage cell line was obtained from ATCC (Manassas, VA) and routinely tested for mycoplasma. The recombinant chimera protein mouse CLEC-2-Fc was produced from pFuse-Fc-CLEC2 stable transfected 293T cell line and shown to bind to podoplanin-positive RAW 264.7 cells using flow cytometry.

### Macrophage phagocytosis and bacterial killing

Phagocytosis: BMDM (untreated or treated with 1 μg/ml of LPS for 24 h, 10^5^ cells/condition) were incubated with Alexa Fluor 488 conjugate-*Escherichia coli* (K-12 strain) BioParticles (3 × 10^6^ particles/per condition) for 60 min at 37 °C. Phagocytosis was assessed using flow cytometry.

Bacterial killing: BMDM (untreated or treated with 1 μg/ml of LPS for 24 h, 10^6^ cells/ml) were incubated with *E.coli* CFT73 live bacteria (10^8^ CFU of bacteria) for 30 or 60 min in the absence of antibiotics at 37 °C. Cells were washed, lysed with water and plated in serial dilutions on LB agar overnight. The number of CFU was counted to measure bacterial killing compared to bacteria alone and subtracted from the total number.

### Data analysis

All data were presented as means ± s.d or s.e.m. The significant difference between two groups was evaluated using a Mann–Whitney *U*-test, and the significance between three groups analyzed using a two-way ANOVA with Tukey’s multiple comparisons test using Prism 7 (GraphPad Software Inc, USA).

### Data availability

All data are available from the corresponding author upon reasonable request.

## Electronic supplementary material

Supplementary Information
