# Supplementary material for: The podoplanin-CLEC-2 axis inhibits inflammation in sepsis
Source: Nat Commun. 2017 Dec 21;8:2239. doi: 10.1038/s41467-017-02402-6 (PMC5740111; doi:10.1038/s41467-017-02402-6)
Supplement: Supplementary file 1 — Supplementary Information [file 41467_2017_2402_MOESM1_ESM.pdf]

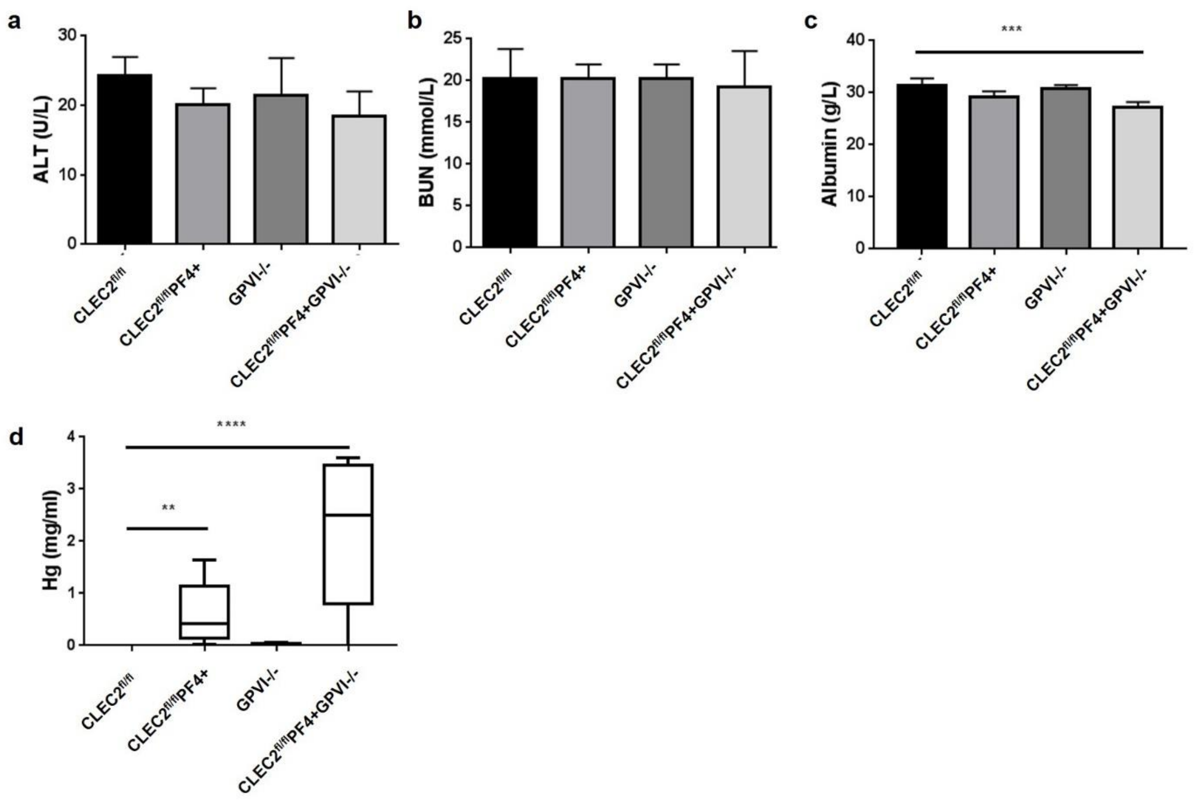

Supplementary Figure 1: Liver and kidney function is not altered in unchallenged mice. (a) Alanine transaminase (ALT), (b) Blood Urea Nitrogen (BUN), and (c) blood albumin levels measured in the blood serum of unchallenged wildtype (CLEC-$2^{fl/fl}$), platelet CLEC-2 deficient (CLEC-$2^{fl/fl}$PF4cre+), GPVI$^{-/-}$ and double knock out (CLEC-$2^{fl/fl}$PF4cre+GPVI$^{-/-}$) mice (n=8). (d) Hemoglobin (Hg) level was measured in the peritoneal cavity of unchallenged mice. Differences between groups were assessed by one-way ANOVA with Tukey's multiple comparison tests. Mean values are shown with s.d. error bars (n=6),**p < 0.01, ***p < 0.001.

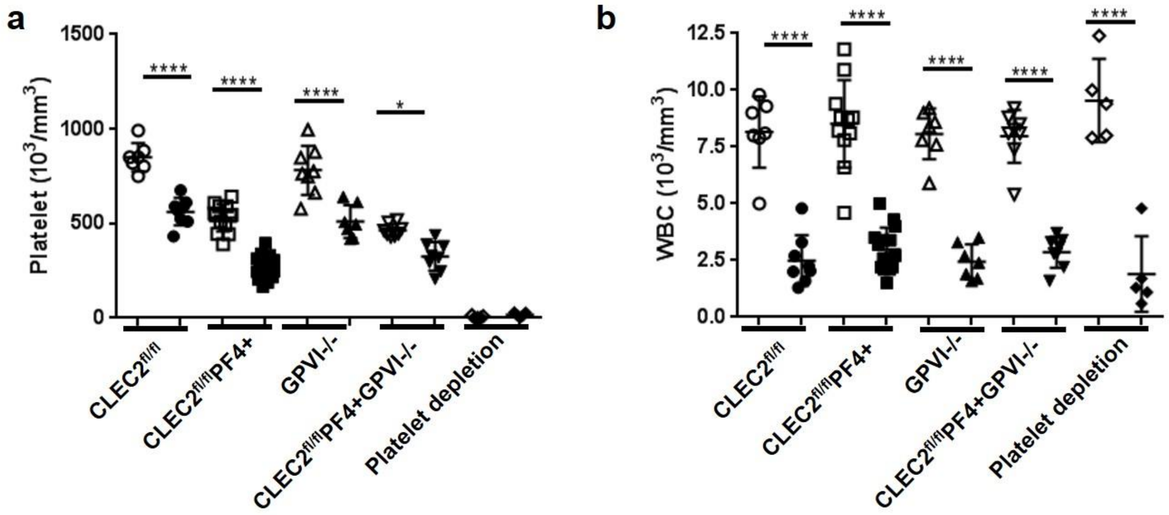

Supplementary Figure 2: LPS induces thrombocytopenia and lymphopenia in mice. Mice were injected with LPS (10 mg/Kg) for 8 h. Blood platelets (a) and white blood cells (WBC) (b) measured before (open symbols) and 8 h post LPS (close symbols) (n ≥ 5, mean values are shown with s.d. error bars). Differences assessed by one-way ANOVA with Tukey's multiple comparison tests. *$p < 0.05$, ****$p < 0.0001$.

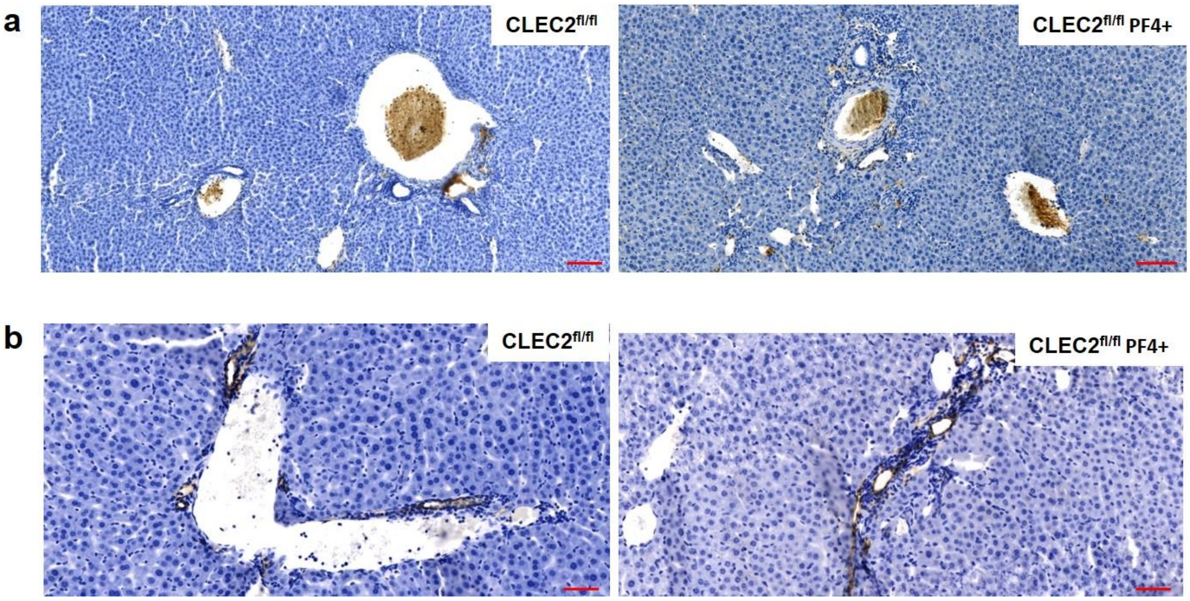

Supplementary Figure 3: Podoplanin is not upregulated in liver vessels 8 h post LPS. Representative paraffin-embedded **l**iver sections from littermate controls or CLEC2$^{fl/fl}$PF4cre+ mice stained for (a) fibrin(ogen) (scale bar = 200μm) or (b) podoplanin (Scale bar = 100μm) (n=5).

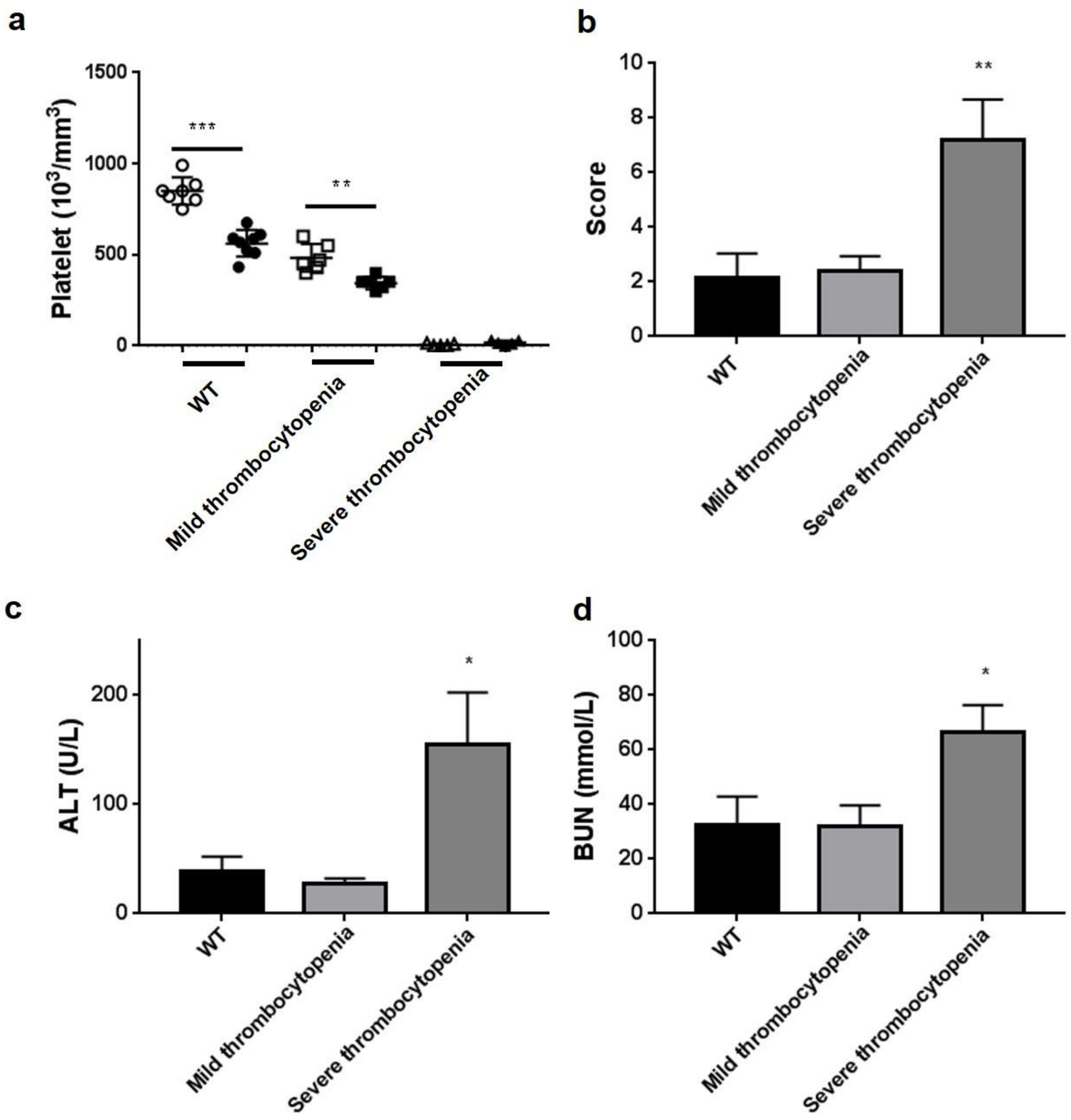

Supplementary Figure 4: Severe thrombocytopenia increases sepsis severity and multiple organ damage. Mice were injected with anti GPIbα antibody (0.3 or 1.5 μg/g to induce mild or severe thrombocytopenia, respectively). Mice were injected with LPS (10 mg/Kg) 24 h post platelet depletion. (a) Platelets count measured before (open symbols) and 8 h post LPS (close symbols) (n≥7). (b) Clinical severity, (c) Alanine transaminase (ALT) and (d) Blood Urea Nitrogen levels measured 8 h post LPS (n=5). Differences between control and other groups were assessed using Mann Whitney U test. Mean values are shown with s.d. error bars *$p < 0.05$, **$p < 0.01$, ***$p < 0.001$.

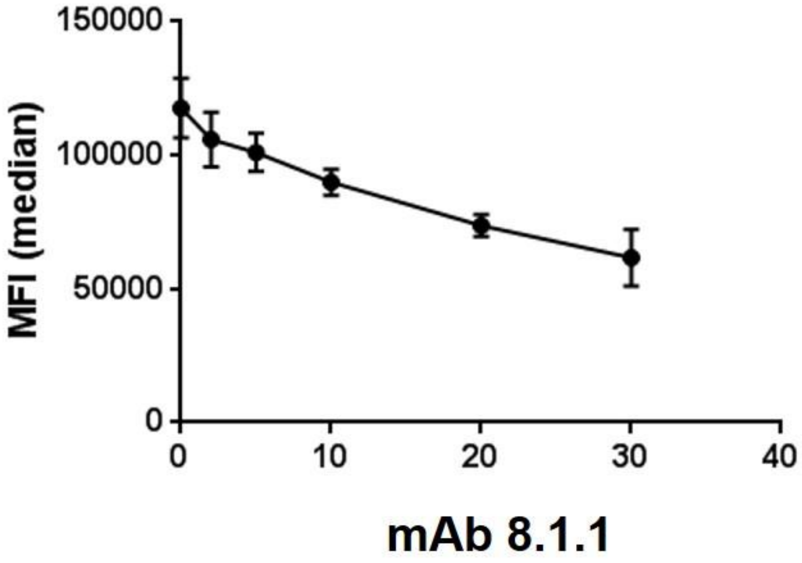

Supplementary Figure 5: The anti-podoplanin antibody mAb 8.1.1 blocks interaction between CLEC-2 and podoplanin. The RAW 264.7 macrophage cell line was stimulated with LPS (1 ng/ml) for 24 h to induce expression of podoplanin. Cells were harvested and Fc blocked then incubated in the presence of increasing concentrations of mAb 8.1.1 for 30 min at 4°C. After washing with PBS, recombinant CLEC-2 was added at 20 µg/ml for 30 min at 4°C. Recombinant CLEC-2 binding to RAW 264.7 macrophages was assessed by flow cytometry using a FITC-labelled anti CLEC-2 antibody (clone 17D9) (n=3). MFI = median fluorescence intensity.

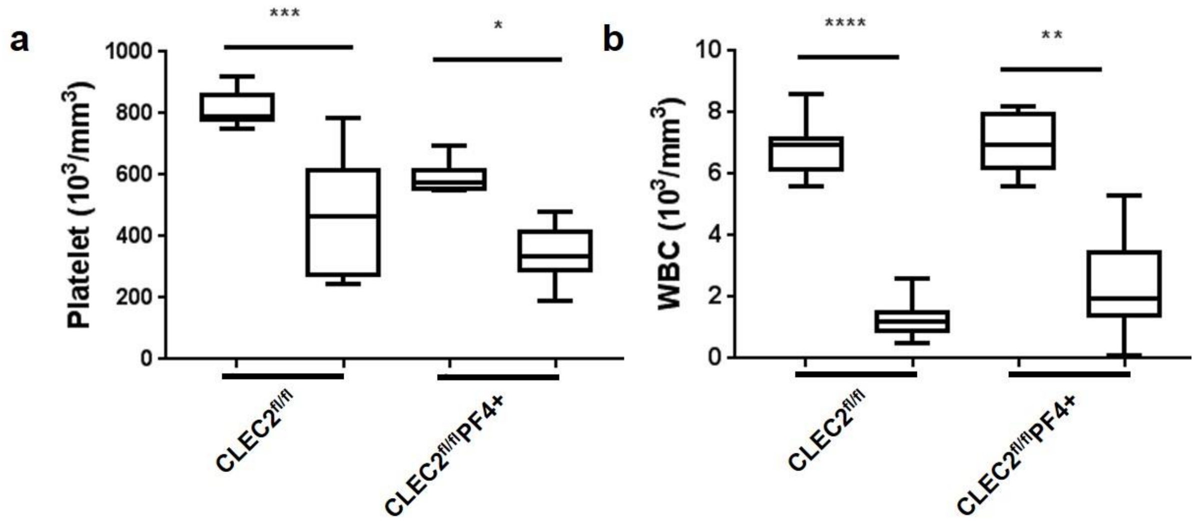

Supplementary Figure 6: Cecal ligation and puncture (CLP) induces thrombocytopenia and lymphopenia in mice. Mice were subjected to CLP for 24 h. (a) Platelet and (b) white blood cells (WBC) count measured in the blood before and 24h post CLP (n=12). Differences assessed by one-way ANOVA with Tukey's multiple comparison tests (Mean ± s.d.). *$p < 0.05$, ***$p < 0.001$, ****$p < 0.0001$.

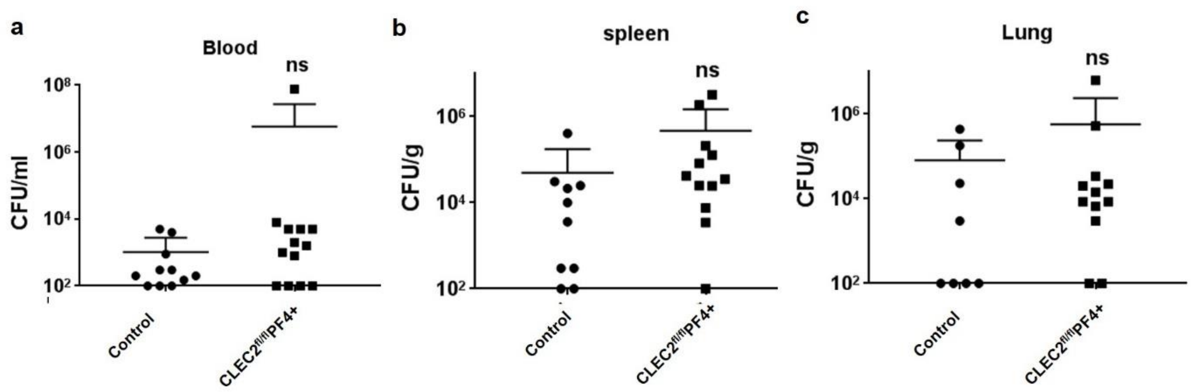

Supplementary Figure 7: CLEC-2 deletion on platelets does not alter bacterial dissemination to the blood, spleen and lung. CLEC2$^{fl/fl}$PF4cre+ or their littermate control mice were subjected to cecal ligation and puncture (CLP) for 24 h. Bacteria in the blood (a) as well as spleen (b) and lung (c) homogenates were assessed post CLP. Colony unit formation (CFU) were counted and adjusted to blood volume or organ weight. Data are means ± s.d (≥7 mice in CLP groups). Differences were assessed using Mann Whitney U test. ns = not significant.

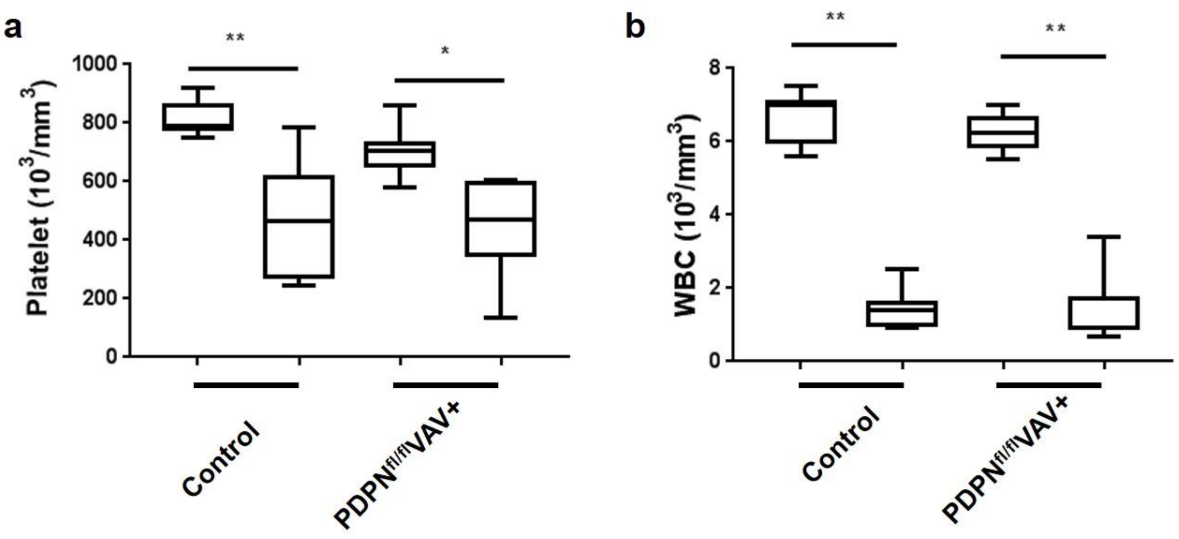

Supplementary Figure 8: Cecal ligation and puncture (CLP) induces thrombocytopenia and lymphopenia in mice with podoplanin-deficient hematopoietic cells. Hematopoietic-specific podoplanin deficient mice (PDPN$^{fl/fl}$VAV+) and littermate controls were subjected to CLP for 24 h (a) Platelets and (b) white blood cells (WBC) count measured post CLP ($n \geq 5$). Differences were assessed using Mann Whitney U test. Mean values are shown with s.d. error bars *$p < 0.05$, **$p < 0.01$, ***$p < 0.001$.

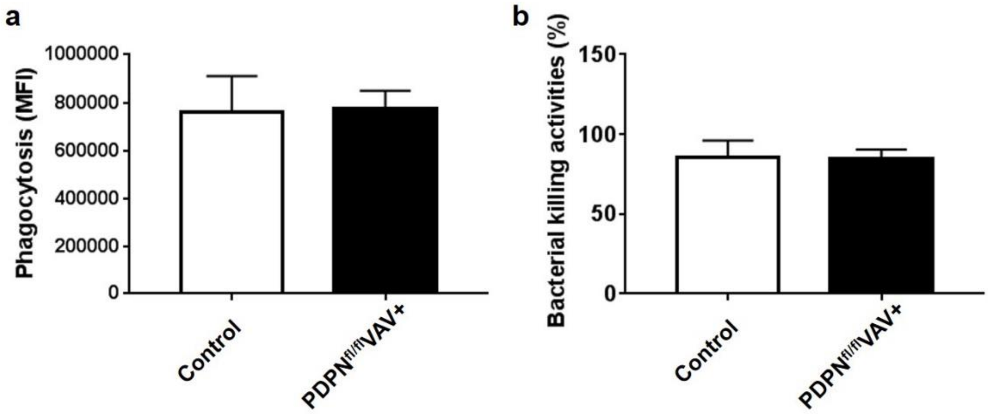

Supplementary Figure 9: Podoplanin expression on bone marrow derived macrophages (BMDM) does not alter their phagocytic or bactericidal capacity *in vitro*. BMDM from hematopoietic-specific podoplanin deficient mice ($PDPN^{fl/fl}$VAVcre+) or their littermate control mice were cultured *in vitro* in the presence of LPS. (a) Phagocytosis of alexa-488 conjugated *E. Coli* Bioparticles assessed by median fluorescence intensity (MFI) by flow cytometry. (b) Bactericidal activity assessed using live *E. coli* CFT73 (10^8/1 million macrophage) for 60 minutes under shaking conditions. Bacteria killing capacity was calculated as the number of bacteria remaining compared to bacteria alone. Differences assessed using Mann Whitney U test (n=3, mean± s.e.m.).

Supplementary Table 1: Systemic clinical score to assess the severity of sepsis in LPS-mediated sepsis

Appearance	0- Coat is smooth<br>1- Patched of piloerection<br>2- Piloerection<br>3- Piloerection with intermittent hunched posture
Motor Activity	0- Normal activity<br>1- Reduced activity<br>2- Activity is impaired
Response to stimulation	0- Normal response to auditory stimulus or touch<br>1- No response to auditory stimulus, normal response to touch<br>2- No response to auditory stimulus, slow response to touch<br>3- No response to auditory stimulus, no response to touch
Eyes	0- Open<br>1- Eyes not fully open, possibly with secretion<br>2- Eyes half closed, possibly with secretion
